# 

**Published:** 1876-05

**Authors:** 


### Contents of May Number.

### ORIGINAL.

ART. I.—Physiological Considerations in Transfusion of Blood. By C. George, M. D., .... 361
ART. II. Medical Society of the County of Albany. Semi-Monthly Meeting, February 23d, 1876, .... 371
ART. III.—Operative Treatment of Pleuritic Effusions. By Herman Mynter, M. D., .... 377
ART. IV.—Abstract of the Proceedings of the Buffalo Medical Association, March 7th, 1876, .... 385

### MISCELLANEOUS.

The Operative Treatment of Pleuritic Exudations, .... 389
Non-idenity of Croup and Diphtheria as shown by their Pathological and Histological Anatomy, .... 392

### EDITORIAL DEPARTMENT.

Preliminary Education of Medical Students, .... 396
Sanitary Condition of Philadelphia, .... 397
Meetings of Medical Societies, .... 399
BOOKS REVIEWED:
Hospital Plans, .... 400
Books and Pamphlets Received, .... 400

THE BUFFALO

